# Extreme exercise in males is linked to mTOR signalling and onset of amyotrophic lateral sclerosis

**DOI:** 10.1093/brain/awaf235

**Published:** 2025-06-18

**Authors:** David O’Brien, Elham Alhathli, Ceryl Harwood, Debarati Bhattacharya, Kriti Gupta, Thomas Julian, Marcel Weinreich, Ryan J H West, Dennis Wang, Ross P Byrne, Russell L McLaughlin, Joanne Wuu, Michael Benatar, Johnathan Cooper-Knock, Pamela J Shaw

**Affiliations:** Sheffield Institute for Translational Neuroscience (SITraN), University of Sheffield, Sheffield S10 2HQ, UK; NIHR Sheffield Biomedical Research Centre, Sheffield S10 2JF, UK; Sheffield Institute for Translational Neuroscience (SITraN), University of Sheffield, Sheffield S10 2HQ, UK; Faculty of Nursing, Taif University, Taif 26571, Saudi Arabia; Barnsley Hospitals NHS Foundation Trust and Barnsley Metropolitan Borough Council, Barnsley S75 2EP, UK; Sheffield Institute for Translational Neuroscience (SITraN), University of Sheffield, Sheffield S10 2HQ, UK; Sheffield Institute for Translational Neuroscience (SITraN), University of Sheffield, Sheffield S10 2HQ, UK; Manchester Centre for Genomic Medicine, Manchester University, Manchester M13 9WL, UK; Department of Clinical Neurobiology at the German Cancer Research Center (DKFZ), Heidelberg University, Heidelberg 69120, Germany; Sheffield Institute for Translational Neuroscience (SITraN), University of Sheffield, Sheffield S10 2HQ, UK; National Heart and Lung Institute, Imperial College London, London SW3 6LY, UK; Complex Trait Genomics Laboratory, Smurfit Institute of Genetics, Trinity College Dublin, Dublin D02 PN40, Ireland; Complex Trait Genomics Laboratory, Smurfit Institute of Genetics, Trinity College Dublin, Dublin D02 PN40, Ireland; ALS Center and the Department of Neurology, University of Miami, Miami, FL 33136, USA; ALS Center and the Department of Neurology, University of Miami, Miami, FL 33136, USA; Sheffield Institute for Translational Neuroscience (SITraN), University of Sheffield, Sheffield S10 2HQ, UK; NIHR Sheffield Biomedical Research Centre, Sheffield S10 2JF, UK; Sheffield Institute for Translational Neuroscience (SITraN), University of Sheffield, Sheffield S10 2HQ, UK; NIHR Sheffield Biomedical Research Centre, Sheffield S10 2JF, UK

**Keywords:** exercise, amyotrophic lateral sclerosis (ALS), gene–environment interaction, mammalian target of rapamycin (mTOR) signalling

## Abstract

Amyotrophic lateral sclerosis (ALS) is thought to be caused by interaction between genetic and environmental factors leading to motor neuron (MN) degeneration. Physical exercise has been linked to ALS but controversy remains. A key question is to determine which individuals might be at risk of exercise-associated ALS, because unnecessary avoidance of exercise could be harmful.

We implemented complementary strategies including Mendelian randomization (MR) and multiple questionnaire-based measures of physical exercise in different cohorts. We include a prospective study involving UK Biobank participants where we could test for a relationship between exercise and the timing of future ALS symptom onset. To interrogate the molecular basis of our observations we performed a genetic association study of ‘extreme’ exercise, equivalent to >6 h of strenuous exercise or >12 h of any leisure-time exercise per week.

Our data suggest that the link between increased physical exercise and ALS is particularly important for males who perform the most activity; with no evidence of a link in females. We determined that extreme exercise in males is associated with loss-of-function genetic variants within a number of mammalian target of rapamycin (mTOR) signalling genes that are also differentially expressed in ALS spinal cord.

Activity-induced mTOR signalling has been shown to selectively benefit MN. Therefore, our findings could imply that moderate exercise is neuroprotective via enhanced mTOR signalling, but extreme exercise in men is associated with neurotoxicity and ALS via a failure of this mechanism. There was no significant overlap between genes associated with extreme exercise and those associated with ALS risk, consistent with a true gene–environment interaction rather than a shared genetic basis. We are not yet able to make individual-level recommendations regarding exercise and risk of ALS, but our conclusions should provide focus for future investigation.

## Teaser

Amyotrophic lateral sclerosis (ALS) has been linked to physical exercise, but it is unclear who may be at risk and through what mechanism. O'Brien *et al.* demonstrate a link between ALS and extreme exercise in males which is potentially mediated via failed mTOR signalling at the neuromuscular junction.

**See Talbot and Thompson (https://doi.org/10.1093/brain/awaf310) for a scientific commentary on this article.**

## Introduction

Amyotrophic lateral sclerosis (ALS) is an incurable neurodegenerative disease characterized by the progressive injury and death of motor neurons (MN). The majority of ALS has been proposed to result from gene–environment interactions.^1^ Identification of these interactions could lead to strategies that aim to prevent ALS. Environmental risk factors are not well understood, but a body of observational data supports the hypothesis that strenuous physical exercise is a contributor to ALS risk.^2,3^ However, this link has also been disputed; for example a recent large prospective study concluded that exercise may be protective against risk of ALS^4^ although the authors acknowledged that they did not specifically study the most extreme exercisers.^5^ We suggest that a key task is to determine which individuals, if any, are at risk of exercise-associated ALS.

One proposal that may explain apparently contradictory observations is that ALS risk is not related to all physical activity, but only to very frequent or strenuous physical exercise. Consistent with this, we^2^ and others^6^ have highlighted the higher incidence of ALS in professional athletes. Physical activity might also have different biological effects in males and females,^7^ and we therefore hypothesize that the relationship between ALS and exercise might differ based on sex.

Here, we set out to explicitly investigate the link between the dose of physical exercise and risk of ALS separately in males and females. We have combined complementary strategies—Mendelian randomization (MR) and questionnaire-based measures, including in a prospective study. Two-sample MR does not require measurement of exercise and ALS incidence in the same individuals^8^ and thereby can more easily achieve very large sample sizes. Moreover, MR avoids selection bias which potentially confounds questionnaire-based studies. However, because MR relies on genetic liability to exercise/ALS rather than a direct measurement, it incorporates some unmeasurable bias.^8^ In contrast, questionnaires are a relatively direct measure and thus complement the MR approach. In particular, the Historical Adulthood Physical Activity Questionnaire (HAPAQ) is a measure of lifetime physical activity which has been validated against objective measurement of historical physical activity.^9^ UK Biobank (UKB)^10^ includes a different questionnaire-based measure of physical exercise but offers the unique opportunity to perform a prospective study, as within this population-scale dataset there are currently 430 individuals who were asymptomatic at enrolment when they completed an exercise questionnaire, but later developed ALS.

A potentially important concept is that exercise itself may not be causally related to ALS but instead both exercise and ALS may share a common genetic basis. In this scenario, a person who carries a genotype associated with strenuous physical exercise may be at increased risk of developing ALS, even if they do not actually perform any exercise, because their genetic background is responsible for the elevated disease risk. ALS has a rare variant architecture^11^ and therefore, to address this possibility, we performed a rare variant genetic association analysis of extreme physical activity in the UKB for comparison with rare genetic variants associated with risk of ALS.

**Figure 1:**
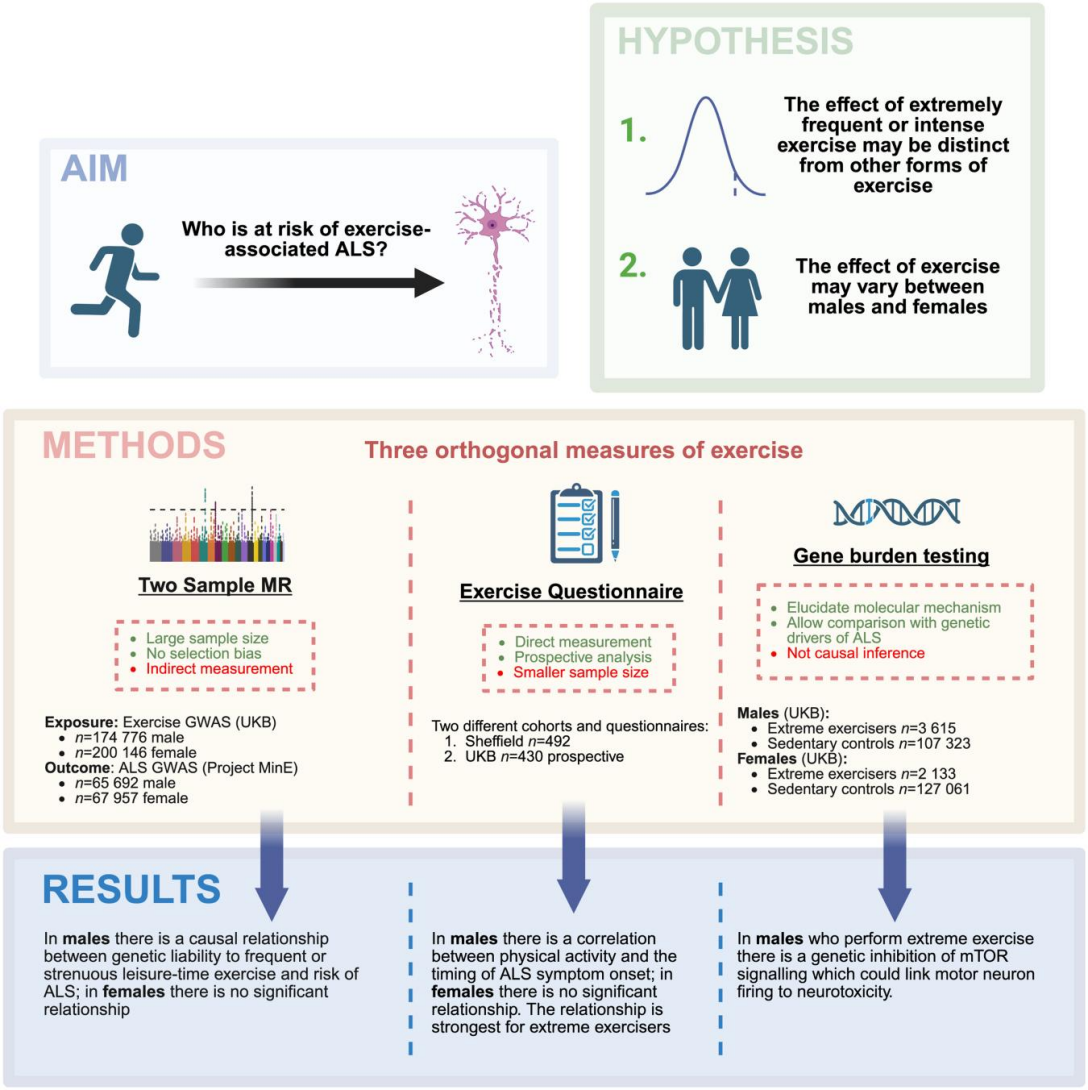
**Approach to identify those at risk of exercise-associated ALS.** We hypothesized that exercise-associated ALS may be determined by frequent or strenuous exercise in a sex-specific manner. To investigate this, we implemented a set of three complementary strategies including two-sample Mendelian randomization (MR) utilizing genetic liability to exercise; and multiple different questionnaire measures of exercise, including a prospective study of UK Biobank (UKB) participants where we could test for a relationship between exercise and the timing of future ALS symptom onset. Finally, to gain molecular-level insight, we examined rare genetic variants associated with an extreme exercise phenotype in males and females separately. ALS also has a rare variant architecture^11^ and one consequence of this final analysis, is that we could determine whether a common set of rare genetic variants predisposes to both extreme exercise and ALS. Created in BioRender. Cooper-Knock, J. (2025) https://BioRender.com/j09u606. ALS = amyotrophic lateral sclerosis; GWAS = genome-wide association study.

Across all cohorts we find convergent evidence for a link between higher frequency or intensity of physical activity in males and younger age of ALS symptom onset. We provide evidence that this association is driven by males who perform the most exercise. To investigate this, we performed a genetic study of the top 5% of exercisers in the UKB, which we defined as ‘extreme’, equivalent to >6 h of strenuous exercise or >12 h of any leisure-time exercise per week. We identified an excess of loss-of-function mutations within mammalian target of rapamycin (mTOR) signalling genes in the male extreme exercise group. mTOR is a protein kinase that functions in intracellular signalling, and has been implicated in the regulation of catabolism and anabolism, including the balance of cell proliferation and cell survival in response to nutrient availability.^12^ Activation of mTOR signalling has been associated with a neuroprotective response triggered by MN activity^13^ thereby offering a mechanism through which exercise might be neuroprotective. We suggest that this protective mechanism is genetically inhibited in a proportion of male extreme exercisers such that exercise predisposes to neurotoxicity and risk of ALS. Consistent with our hypothesis the same mTOR signalling genes are differentially expressed in ALS spinal cord. Our experimental approach and results are summarized in Fig. 1.

An important point is that our analyses do not permit individual-level conclusions and so we cannot offer individualized recommendations for safe physical activity. Our findings should help to focus future investigation of exercise-associated ALS on males performing extreme exercise, but we are cognisant that unnecessary reduction of exercise could be detrimental to general health.^14^

## Materials and methods

### Sex-specific genome-wide association study for frequent or intense physical exercise

Sex-specific genome-wide association study (GWAS) for physical exercise was performed using UKB data; we used the strenuous sport and other exercise (SSOE) definition as in Klimentidis *et al*.,^15^ which includes all physical exercise that is not occupational or performed as a by-product of other activities of living such as cleaning or childcare, i.e. leisure-time physical exercise. As discussed previously, this captures activity that is more likely to be anaerobic and involve MN subtypes that are most vulnerable to ALS.^16^ As part of the UKB study, self-reported levels of physical activity were quantified via a touchscreen questionnaire, similar to the ‘International Physical Activity Questionnaire’ (IPAQ).^17^ This study used responses from the first question ‘Did you spend any time doing the following over the past 4 weeks?’, in addition to follow-up questions to measure the frequency and duration of ‘strenuous sports’ and ‘other exercises’. The different options for the first questions were: ‘walking for pleasure’, ‘other exercises’, ‘strenuous sports’, ‘light do-it-yourself (DIY, i.e. the activity of decorating or repairing a home)’, ‘heavy DIY’, ‘none of the above’ and ‘prefer not to answer’. To capture all options related to leisure-time exercise we compared individuals spending 15–30 min or more during ‘strenuous sports’ or ‘other exercises’ (SSOE) 2–3 days/week or more, against controls who did not report any time performing ‘strenuous sports’ or ‘other exercises’ in the previous 4 weeks. The male-specific GWAS included 62 432 frequent or intense exercisers versus 112 344 controls; the female-specific GWAS included 67 983 frequent or intense exercisers versus 132 163 controls.

When performing the GWAS, we only included imputed single nucleotide polymorphisms (SNPs) from the Haplotype Reference Consortium. Sample exclusion criteria included mismatches between self-reported and genetically inferred sex, abnormally high heterozygosity and a >5% missing rate. SNP exclusions were based on low minor allele frequency (<0.1%), low minor allele count (<20), high missingness (>1.5%), low imputation quality (info <0.4) and deviation from Hardy–Weinberg equilibrium (*P* < 1 × 10^−6^).

Male- and female-specific GWAS were carried out using the Swiss Army Knife tool and REGENIE^18^ as implemented in the UKB DNA Nexus platform. Age, the first 10 genomic principal components, the season (month) at the centre visit, and the enrolment centre attended were used as covariates. SNPs were linkage disequilibrium (LD) pruned to include only independent SNPs (*r*^2^ > 0.4).

### Sex-specific GWAS for ALS risk

We used a sex-specific ALS GWAS from Project MinE (https://www.projectmine.com/) reported previously.^19^ The male-specific GWAS included 15 547 ALS cases and 50 145 controls and the female-specific GWAS included 10 895 ALS cases and 57 062 controls. This cohort is independent of the exposure GWAS used to measure physical exercise.

### Two-sample MR for the effect of physical exercise on ALS risk

Genetic variants or SNPs associated with ‘exposure’ to the SSOE trait were used as genetic instruments; we employed a *P*-value cut-off of <5 × 10^−5^ to select SNPs based upon our previous work.^16^ Independent SNPs were clumped using a stringent cut-off of *R* < 0.001 within a 10 000 kb window in the European reference panel. For clumped SNPs in LD, the SNP with the lowest *P*-value was retained. When an exposure SNP was unavailable in the outcome dataset, a proxy with a high degree of LD (*R*^2^ > 0.9) within a European reference population was identified. SNP effects on outcomes (ALS risk) and exposures were harmonized so that the beta values were based on the same alleles. In order to reduce the risk of errors due to strand issues, palindromic alleles with minor allele frequency (MAF) >0.42 were excluded from the analysis.

MR was performed using a multiplicative random-effects inverse-variance weighted (IVW) estimate for significance testing, as this method has the greatest statistical power^20^; this measure is accurate under the assumption of limited balanced pleiotropy. In order to increase confidence in the IVW results we performed a series of robust MR measures and sensitivity analyses. We used an *F*-statistic to measure the strength of the association between instrumental SNPs and the exposure of interest. An *F*-statistic >10 indicates that a SNP-derived estimate has a bias of <10% of its intragroup variability and signifies an acceptable instrument. Pleiotropy occurs between SNPs where the difference in effect size for the exposure is not proportional to the difference in effect size for the outcome, and is usually due to a violation of one of the key assumptions underlying MR, the assumption that instrumental SNPs should be associated with the outcome only through the exposure.^8^ To account for pleiotropy, we removed SNPs where the *P*-value for the association with the outcome was lower than for the association with the exposure of interest. The MR-Egger intercept test was also used to identify directional horizontal pleiotropy. We also used Cochran's Q test (*P* > 0.05) as a sensitivity measure to detect heterogeneity indicating pleiotropy. Moreover, radial-MR^21^ was used to remove statistically significant outlier SNPs before conducting any other statistical test. The *I*^2^ statistic was used to measure the heterogeneity between variant-specific causal estimates, with a low *I*^2^ indicating bias toward the null hypothesis.^22^ A leave-one-out (LOO) analysis was applied to identify results where one or more SNPs exert a disproportionate effect. TwoSampleMR (version 0.5.6, https://github.com/MRCIEU/TwoSampleMR), Mendelian Randomization (version 0.5.1, https://amymariemason.github.io/MR/) and RadialMR (version 1.0, https://github.com/WSpiller/RadialMR) R packages were used for all MR analyses.

### Questionnaire measurement of historical physical exercise using HAPAQ

Data were analysed from a previous questionnaire study of ALS cases and healthy controls undertaken by Harwood *et al*.^23^ Between 2009 and 2013, this study recruited 175 ALS cases (median age 65 years, 62% male, 68% El-Escorial clinically definite or probable, 70% limb onset) and 317 healthy controls (median age 64 years, 62% male) from the North of England. ALS cases were recruited from a tertiary clinic within 6 months of diagnosis, and age-, sex- and geographical location-matched controls were recruited via invitation from their general practitioners. To quantify historical physical activity, the study team administered the HAPAQ, which has been validated against objective measurements of physical activity.^9^

From the results of the HAPAQ questionnaire, durations of various types of physical activity were calculated for each decade of life. Inputting the metabolic equivalent value (MET) for each activity undertaken then enabled conversion into a value of physical activity energy expenditure (PAEE).^9,23^ Leisure-time physical exercise, which is equivalent to activity captured by the SSOE measure described above, was derived by summing the physical activity for all strenuous sport and exercise, and more casual sport and exercise. The relationship between sex-specific historical leisure-time physical activity and the age of ALS onset was calculated by multivariate Cox regression with inclusion of covariates including employment/student/professional sportsperson status, time off-work through sickness, any children, use of motor vehicles, pets and house type. We isolated exercise between the ages of 20 and 39 years to avoid confounding by reducing physical exercise with age.^23^ We excluded any ALS cases with symptom onset earlier than 45 years old to account for a premorbid period potentially impacting upon activity levels.

### Questionnaire measurement of physical exercise in UKB participants who later developed ALS

Physical activity in UKB participants was quantified in MET minutes. One MET corresponds to an activity where 1 kcal per kilogram per hour is consumed. A MET minute corresponds to one minute of that activity. MET minutes per week is thus a measure of the amount of energy expended per week quantified in multiples of this unit. UKB includes a measure of total activity in MET minutes based on reported activity at the time of enrolment.^10^ The relationship between physical activity in MET minutes per week, and age of ALS symptom onset or time to ALS onset from UKB recruitment, was calculated by multivariate Cox regression with inclusion of current age as a covariate. In order to plot Kaplan–Meier curves, we divided participants into four equal-sized percentiles basd upon their MET minutes per week of physical activity. The first quantile largely consisted of individuals with zero recorded activity and therefore we compared the second and fourth quantiles.

To perform a sensitivity analysis we ranked individuals by MET minutes per week of physical activity. We then repeated the Cox regression tests but with removal of the top 20 individuals by activity rank. Further repeat tests removed 20 progressively lower ranked individuals; in each test the 20 individuals overlapped the cohort removed in the previous test by 19 individuals. After tests had been performed with removal of all individuals at least once, we then calculated the Pearson correlation between the mean MET minutes per week of physical activity in the removed subset, and the age of ALS symptom onset in the remaining individuals. The idea was to determine whether individuals performing more or less physical activity disproportionately affected the results of our analysis. If all individuals contributed equally then we might expect no significant correlation.

A caveat is that the UKB does not record the precise date of ALS diagnosis but instead the date when the diagnosis was reported to the UKB, which may not be equivalent but should not precede the actual date of diagnosis. Moreover, we expect that the difference between the recorded date and the actual date of diagnosis is independent of the amount of exercise performed.

### Rare variant gene burden testing in extreme exercisers

To isolate individuals performing extreme quantities of physical exercise in the UKB we selected the top 5% of exercisers within the SSOE category described above; this equated to those who performed more than 6 h of ‘strenuous exercise’ or more than 12 h of other leisure-time exercise per week. More precisely we selected males or females who performed ‘strenuous exercise’ everyday for more than 15–30 min; and ‘strenuous exercise’ 4–5 times a week for more than 1.5–2 h per session; and ‘strenuous exercise’ 2–3 times a week for more than 3 h per session; and ‘other exercise’ everyday for more than 1.5–2 h per session; and ‘other exercise’ 4–5 times a week for more than 2–3 h per session. The total number of males who met these criteria was 3615; the total number of females who met these criteria was 2133. This group was used in a rare variant gene burden analysis. As a control we compared against males (*n* = 107 323) or females (*n* = 127 061) who reported zero hours of ‘strenuous exercise’ or other leisure-time exercise per week.

Rare variant analysis was conducted using REGENIE^18^ within the UKB DNA Nexus platform. Variant quality control (QC) was performed as previously described.^24^ We considered only variants that were rare (MAF < 1%) and loss-of-function (LOF) as defined by nonsense or splice-site variants. A significant association of LOF variants within a particular gene, and performance of extreme exercise was calculated by SKATO^25^ using age, genotyping method, first 10 genomic principal components, enrolment centre and month of enrolment as covariates; as a cut-off for significance we used false discovery rate (FDR) < 0.05.

Overlap between the genetic basis of extreme exercise and ALS either ALS risk genes, or genes differentially expressed in ALS spinal cord,^26^ was assessed by the Fisher's exact test. Gene expression analysis in ALS cervical spinal cord is previously described.^26^ In brief: expression counts were Trimmed Mean of M-values (TMM) normalized and significance was calculated using limma-voom^27^ using sex, library preparation method, contributing site, RNA quality and the first five expression principal components as covariates.

## Results

### MR links genetic susceptibility to frequent or strenuous leisure-time physical activity, to risk of ALS

Previously we have used MR to provide evidence for a causal association between genetic liability to frequent or strenuous leisure-time exercise on the one hand, and the risk of ALS on the other.^16^ In view of a recent prospective population study identifying sex differences in the protective effect of exercise on the risk of ALS,^4^ we have repeated our analysis in males and females separately. Using data from UKB, we performed sex-specific GWAS for frequent or strenuous leisure-time exercise^15,16^ to identify genetic instruments that infer liability to frequent or strenuous exercise. Next we tested for a causal effect of this genetic liability to exercise on disease occurrence using sex-specific GWAS of ALS.^19^ Importantly the ALS GWAS was carried out in an independent cohort (Project MinE), which is necessary to avoid false positive MR results.^8^

**Figure 2:**
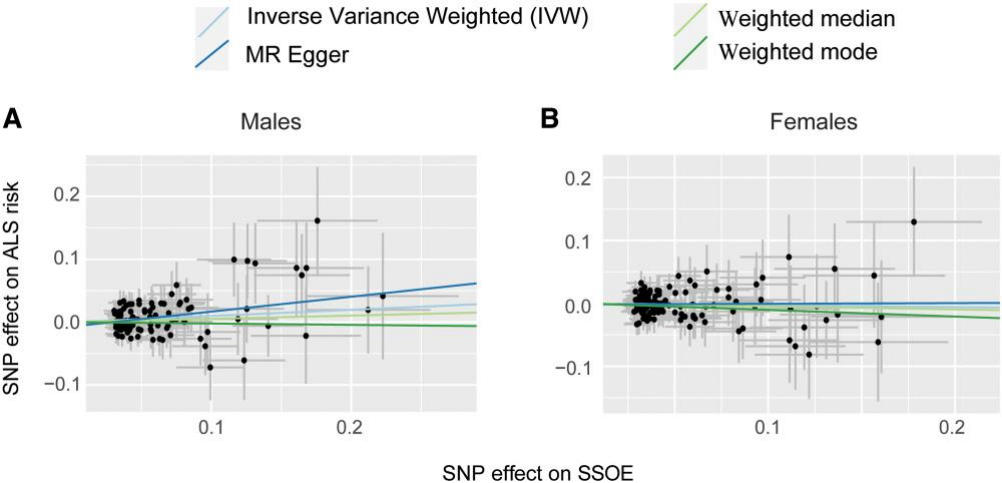
**Two-sample Mendelian randomization (MR) tests for a causal effect of frequent or strenuous leisure-time exercise (SSOE) on risk of ALS**. Scatter plots demonstrate a significant association of exercise with risk of ALS, including in robust MR tests, for (**A**) males, but not for (**B**) females. Each point represents the effect size (beta) and standard errors for each SNP–outcome relationship. See the ‘Materials and methods’ section for details of SSOE. ALS = amyotrophic lateral sclerosis; SNP = single nucleotide polymorphism; SSOE = strenuous sport and other exercise.

In males there was a significant causal relationship between genetic liability to frequent or strenuous leisure-time exercise and risk of ALS (IVW *P* = 0.01, *beta* = +0.1, standard error = 0.04; Fig. 2A and Table 1); this was also significant in the MR Egger test and there was no evidence that the test was invalidated by weak instruments or pleiotropy (Table 1). However, in females there was no significant link between genetic liability to frequent or strenuous leisure-time exercise and risk of ALS (IVW *P* = 0.418, *beta* = −0.03, standard error = 0.04; Fig. 2B and Table 1).

**Table 1:** Test statistics and sensitivity measures for MR analysis of the effect of frequent or SSOE on risk of ALS in males and females

Test	Female SSOE	Male SSOE
IVW *P*-value	0.42	**0.01**
IVW beta	−0.03	0.1
Weighted median *P*-value	0.49	0.4
Weighted median β	−0.05	0.05
Egger *P*-value	0.94	**0.04**
Egger β	0.01	0.24
Weighted mode *P*-value	0.55	0.9
Weighted mode β	−0.1	−0.02
Mean *F* test	19.9	19.8
IVW Cochran's *Q* test *P*-value	0.99	0.99
Radial MR outlier SNPs	9	9
Egger intercept test	0.77	0.19
*I* ^2^	0.95	0.95
Number of SNPs LOO > 0.05	120	0
Total number of SNPs	120	112

Nominally significant *P*-values are in bold. ALS = amyotrophic lateral sclerosis; SNP = single nucleotide polymophism; SSOE = strenuous leisure-time exercise (see the ‘Materials and methods’ section for further information); MR = Mendelian randomization; IVW = inverse variance weighted MR estimate; LOO = leave-one-out.

### Directly measured leisure-time physical exercise is linked to age of ALS symptom onset in males

MR is robust to selection bias which can confound measures of physical exercise, and two-sample MR benefits from very large sample size achieved when combining measurements in different cohorts.^8^ However, our MR test relies on indirectly measuring exercise via associated genetic instruments. To support our findings via a direct measurement, we performed a *post hoc* analysis of a previously published case-control study that utilized a validated questionnaire to quantify historical adulthood physical exercise (HAPAQ).^23^ This analysis included 175 sporadic ALS cases and 317 healthy controls. We hypothesized that, if exercise is linked to risk of ALS, then those individuals who performed more frequent or intense exercise might have an earlier age of ALS symptom onset. Indeed, a previous study of Italian professional footballers noted that ALS disease onset occurred 20.2 years earlier than is typical.^28^

**Figure 3:**
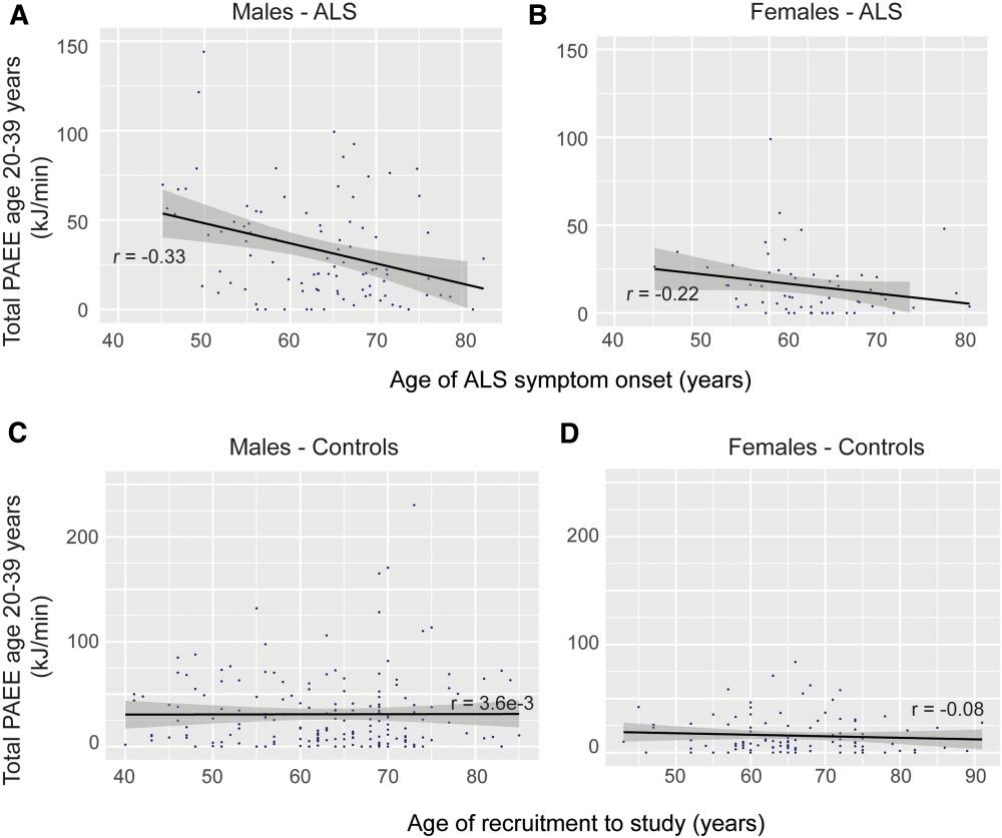
**Higher level of adulthood leisure-time physical activity is associated with younger age of ALS symptom onset in males but not in females**. Historical physical activity was quantified using the HAPAQ^23^ questionnaire. Plots show total leisure-time physical activity energy expenditure (PAEE) for each individual measured in kJ/min, against age of symptom onset for ALS patients (**A** and **B**) and age of study enrolment for controls (**C** and **D**). Males (**A** and **C**) are plotted separately from females (**B** and **D**). For a difference in average leisure-time activity of 100 kJ/min, equivalent to very strenuous exercise in an 80 kg male, there is a 2-fold increase in risk of ALS symptom onset at any given time. A linear regression line is calculated by OLS; shading represents a confidence interval calculated from the standard error of the regression line at each point. ‘r’ indicates the Pearson correlation coefficient. HAPAQ = historical adulthood physical activity questionnaire; OLS = ordinary least squares.

To avoid confounding by age-associated reduction in physical exercise we confined our analysis to exercise performed between 20 and 39 years of age, which is significantly before the typical age of ALS onset.^29^ Our MR study identified a link between frequent or strenuous leisure-time exercise and risk for ALS in males. To isolate comparable levels of physical activity we only considered activity classified as ‘leisure-time physical activity’. After controlling for other factors which could confound the relationship between physical exercise and ALS, including employment, chronic illness, pet ownership and family size, we observed a significant correlation between leisure-time physical activity and age of symptom onset in males (*n* = 92, Cox regression, hazard ratio (HR) = 2 for a difference in average leisure-time physical activity per day of 100 kJ/min, *P* = 0.003; Fig. 3A and ‘Materials and methods’ section). This suggests that a difference in average leisure-time activity equivalent to very strenuous exercise in an 80 kg male, leads to a 2-fold increase in risk of ALS onset at any given time. There was no statistically significant relationship in females (*n* = 58, *P* = 0.2; Fig. 3B). Performing this analysis including all subjects irrespective of sex also revealed a significant relationship (*n* = 150, *P* = 2 × 10^−3^) but including sex as a covariate (*n* = 150, *P* = 1 × 10^−3^) resulted in a lower Akaike information criterion (AIC), indicating an improved model fit while accounting for model complexity (AIC reduced from 1268 to 1211). This is consistent with an effect of sex on the relationship between historical exercise and age of symptom onset irrespective of sample size. As expected, there was no significant relationship between age at study enrolment of controls, and leisure-time physical activity between 20 and 39 years of age (*P* > 0.05; Fig. 3C and D and Supplementary Table 1).

### Prospectively measured physical activity is linked to age of symptom onset of ALS in males

To support our findings, we performed an analysis of the 430 ALS patients within UKB who developed disease after enrolment, and who had completed an exercise questionnaire,^10^ offering an opportunity to perform a prospective analysis (Table 2 and see ‘Materials and methods’ section). Unlike HAPAQ, this questionnaire quantified exercise at a single time point, which has certain disadvantages. For example an analysis of early adulthood physical exercise is not possible and it is difficult to be sure that an ALS prodrome is not artificially depressing measured activity.^5^ However, it is possible to test whether higher levels of physical activity at time of enrolment to UKB are associated with lower age of ALS symptom onset and shorter time from enrolment until symptom onset. This is the opposite to the expected effect of an ALS prodrome, but would be consistent with a causal effect of exercise on ALS onset. We excluded individuals who had developed ALS before enrolment to UKB. Physical activity was quantified as MET minutes per week. One MET corresponds to an activity where 1 kcal per kilogram per hour is consumed and a MET minute corresponds to one minute of that activity. Very strenuous exercise in an 80 kg male corresponds to ∼100 kJ/min or ∼17 MET minutes.

**Table 2:** UK Biobank participants who prospectively developed ALS

Cohort	Number	Mean age at enrolment (mean years ± SD)	Sex (male:female)	Age of ALS symptom onset (mean years ± SD)	Time from enrolment to ALS symptom onset (mean years ± SD)
Prospective ALS	607	60.6 ± 6.7	55:45	68.5 ± 7.6	8 ± 3.7
Prospective ALS with exercise questionnaire	430	60.4 ± 6.7	58:42	68.4 ± 7.6	7.9 ± 3.7

ALS = amyotrophic lateral sclerosis; SD = standard deviation.

**Figure 4:**
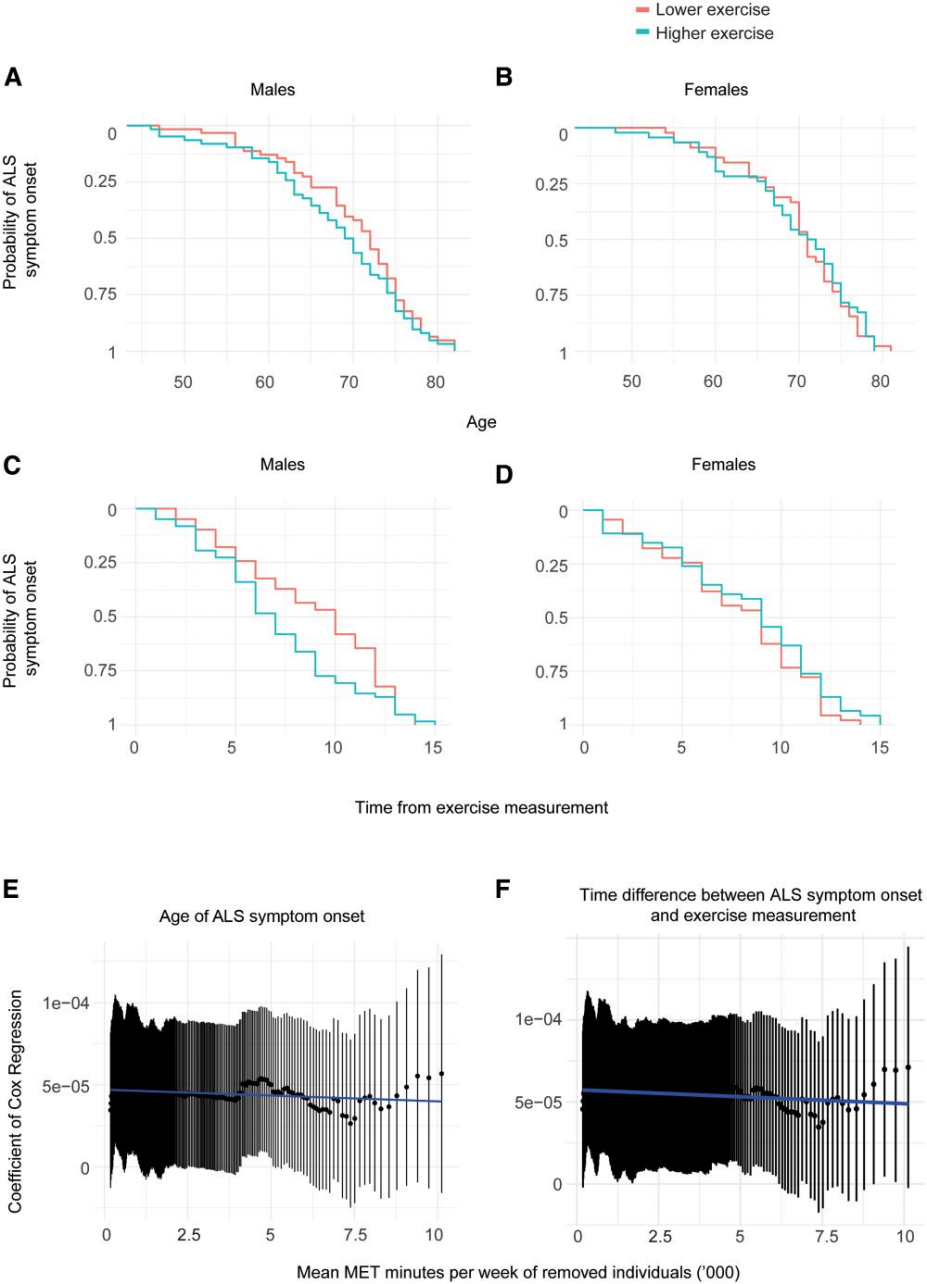
**Prospectively measured physical activity is linked to age of symptom onset of ALS in males.** Utilizing the UKB enrolment questionnaire, total physical activity is quantified in MET minutes per week for participants who later developed ALS. As shown by Kaplan–Meier curves, for males (**A** and **C**) but not for females (**B** and **D**), increased physical activity is associated with earlier age of ALS symptom onset (**A** and **B**) and shorter time from questionnaire to symptom onset (**C** and **D**). Lower and higher levels of physical activity were defined by comparing quartiles of MET minutes per week. We performed a sensitivity analysis whereby we sequentially removed overlapping groups of 20 participants with progressively increasing MET minutes per week of physical activity and repeated a Cox regression test of the association between physical activity and the timing of future ALS symptom onset. The regression coefficient for the effect of physical activity (*y*-axis) is plotted against the mean physical activity of removed participants (*x*-axis) with 95% confidence intervals and line of best fit for age of ALS symptom onset (**E**), and the time interval from questionnaire to symptom onset (**F**). The regression coefficient is negatively correlated with the mean value of physical activity performed by the removed subset, suggesting that the observed association between physical activity and ALS symptom onset is disproportionately driven by individuals who perform the highest levels of physical activity. See the ‘Materials and methods’ section for further details. ALS = amyotrophic lateral sclerosis; UKB = UK Biobank; MET = metabolic equivalent value.

Amongst males (Cox regression, *n* = 248, *P* = 0.018, HR = 2 for a difference in total physical activity of 10 000 MET minutes per week), but not females (*P* = 0.13), total physical activity (Methods) was linked to earlier age of ALS symptom onset after controlling for age at enrolment (Fig. 4A and B). Similarly, higher physical activity is significantly correlated with a shorter time between UKB enrolment and ALS symptom onset in males after controlling for age at study enrolment (Cox regression *P* = 0.049, HR = 2 for a difference in total physical activity of 50 000 MET minutes per week) but not females (*P* = 0.08) (Fig. 4C and D). This means that 9 h per week of very strenuous exercise in an 80 kg male (∼100 kJ/min), leads to a 2-fold increase in risk of ALS onset at any given time.

MET minutes per week is a measure of all physical activity whereas our other analyses have focused on leisure-time physical exercise which is more likely to be strenuous. To test whether strenuous exercise is driving the signal observed in this analysis, we conducted a sensitivity analysis whereby we systematically removed a subset of individuals with progressively increasing MET minutes per week of physical activity. The mean quantity of physical activity performed by the removed subset was negatively correlated with the strength of the association between physical activity and age of ALS symptom onset in the remaining individuals (Pearson correlation, *cor* = −0.31, *P* = 1.59 × 10^−6^; Fig. 4E). The same was true for the association between physical activity and the time interval from UKB enrolment to symptom onset (*cor* = −0.27, *P* = 1.84 × 10^−5^; Fig. 4F). We conclude that the observed association between physical activity and ALS symptom onset is disproportionately driven by individuals who perform the highest levels of physical activity. The highest levels of physical activity are likely to overlap with the frequent or strenuous leisure-time exercise, which was the focus of our prior analyses.

### Rare variant analysis of extreme exercise

**Figure 5:**
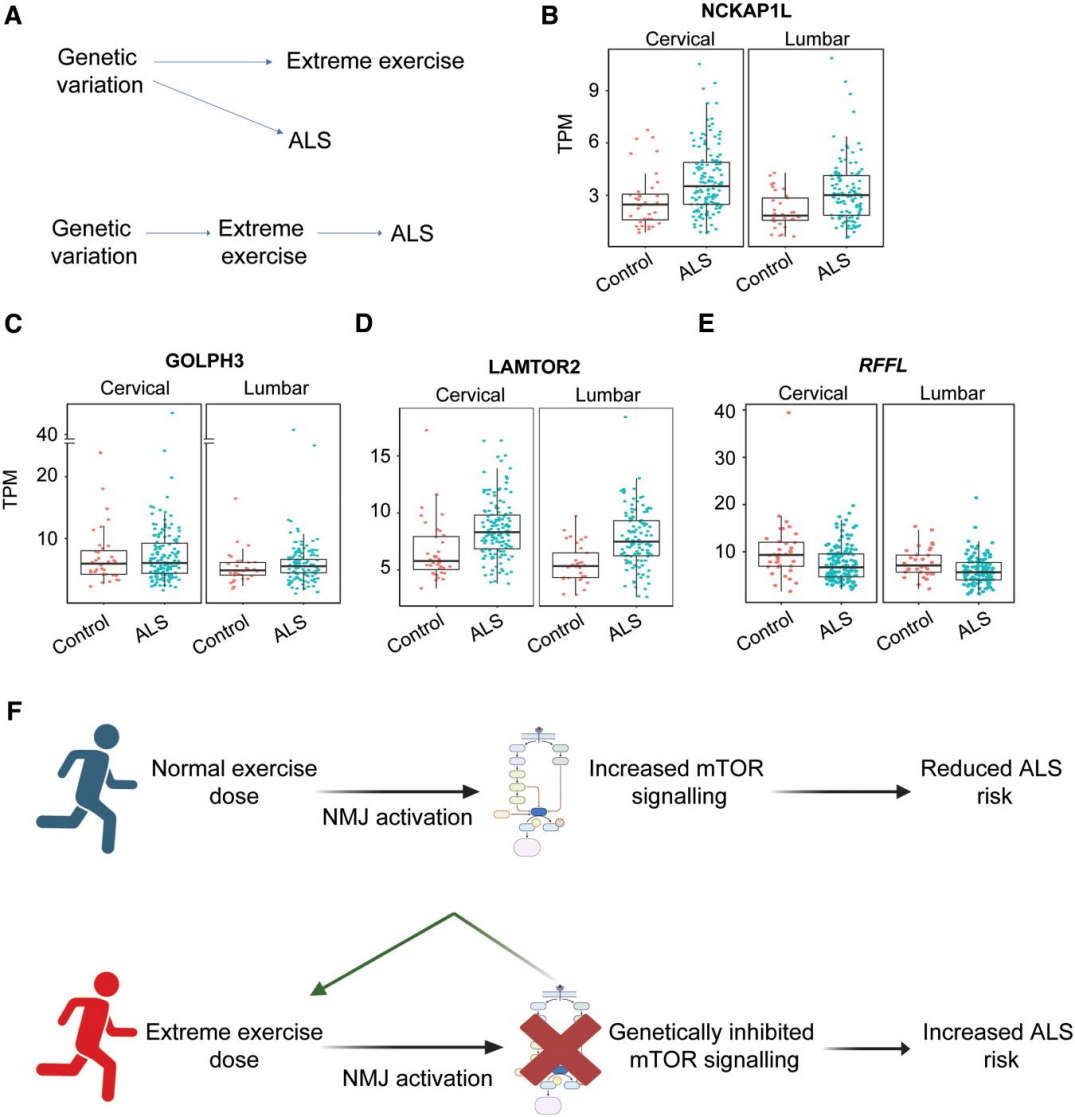
**Rare genetic variant burden testing links extreme exercise to loss-of-function mutations within mTOR signalling genes, which are also differentially expressed in ALS spinal cord**. (**A**) We explored alternative but non-exclusive models for the link between genetic background, extreme exercise and ALS risk. Hypothetically, genetic risk could be linked to exercise and ALS independently (*top*) or genetic drivers of extreme exercise could act on ALS risk only through performed exercise (*bottom*). We did not identify overlap between genes linked to extreme exercise and ALS risk genes suggesting the latter model is correct. Bulk RNA sequencing of ALS (*n* = 154) and control (*n* = 54) spinal cord and analysis of gene expression was previously performed.^26^ Transcript expression is quantified as TPM and plotted against disease status for cervical and lumbar spinal cord segments. Box plots show median and 95% confidence interval of expression. Results are shown for four mTOR signalling genes—(**B**) *NCKAP1L*, (**C**) *GOLPH3*, (**D**) *LAMTOR2* and (**E**) *RFFL*—which are also enriched with loss-of-function mutations in males who perform extreme exercise. TPM indicates transcripts per kilobase million or the number of read counts per length of transcript per million reads mapped. (**F**) Previous studies have suggested that NMJ activation can be neuroprotective via activation of mTOR signalling.^13^ We suggest a model whereby exercise, via activation of mTOR signalling can ‘normally’ protect MN from disease (**F**, *top*). However, genetic inhibition of mTOR signalling prevents this protection while simultaneously predisposing (green arrow) to extreme exercise which is now rendered neurotoxic (**F**, *bottom*). Created in BioRender. Cooper-Knock, J. (2025) https://BioRender.com/zxcqdn5. mTOR = mammalian target of rapamycin; ALS = amyotrophic lateral sclerosis; TPM = transcripts per kilobase million; NMJ = neuromuscular junction; MN = motor neuron.

We have provided evidence that higher levels of physical activity may drive an association between physical exercise and younger age of symptom onset for ALS in males. This could be the result of a gene–environment interaction, i.e. exercise could be harmful to motor neurons (MN) in the presence of a specific genetic background. An alternative explanation is that genetic drivers of extremely strenuous exercise are also genetic drivers of ALS, independent of the actual exercise performed. These alternatives are summarized in Fig. 5A. To explore this hypothesis, we have identified genes where LOF mutations are associated with extreme exercise in males and females (see ‘Materials and methods’ section).

We isolated a group of male extreme exercisers in the UKB, which we defined as males who performed more than 6 h of ‘strenuous exercise’ or more than 12 h of other leisure-time exercise per week (*n* = 3615), which represents the top 5% of exercisers based on the SSOE trait in the UKB. As a control we compared against males who reported zero hours of ‘strenuous exercise’ or ‘other leisure-time exercise’ per week (*n* = 107 323). We tested for differences in the distribution of rare (MAF < 1%) LOF genetic variants across all coding genes. After stringent multiple testing correction, LOF of 108 genes were associated with performance of extreme exercise (Firth logistic regression, FDR < 0.01; Table 3). We did not observe a significant overlap between known ALS risk genes^30^ (Supplementary Table 2) and the 108 genes that achieved significance in our analysis (Fisher's exact test, *P* = 0.3; Fig. 5A). However, the 108 genes significantly overlap with a set of genes that are differentially expressed in ALS compared with control cervical spinal cord (Fisher exact test, *P* = 0.03, overlap of 29 genes).^26^ The 29 overlapping genes (Table 3) are significantly enriched with genes linked to ‘Positive regulation of TOR signalling’ (GO:0032008) (Fisher exact test, adjusted *P* = 0.02) and ‘Regulation of TOR signalling’ (GO:0032006) (adjusted *P* = 0.04). Activation of ‘mTOR signalling’ is thought to be protective to MN^13^ and therefore genetic inhibition of this pathway in the context of extreme exercise may be detrimental to MN health.

**Table 3:** Genes enriched with loss-of-function mutations in males who perform extreme exercise, which are also differentially expressed in ALS versus control cervical spinal cord

Gene	Genetic LOF association with extreme exercise		Gene expression in ALS versus control cervical spinal cord	
	OR	FDR	FC	FDR
*ADCY6*	5.88	3.41 × 10^−3^	0.83	7.81 × 10^−5^
*AZIN2*	5.79	3.35 × 10^−3^	0.66	3.78 × 10^−5^
*BBS12*	3.94	6.13 × 10^−3^	1.26	1.73 × 10^−3^
*C2orf68*	7.12	1.26 × 10^−3^	0.91	2.76 × 10^−3^
*CCR10*	15.43	2.21 × 10^−3^	1.41	2.85 × 10^−4^
*CD14*	5.79	9.22 × 10^−3^	2.08	2.83 × 10^−5^
*CMKLR1*	6.17	9.22 × 10^−3^	1.63	8.92 × 10^−6^
*CYP2S1*	5.14	3.68 × 10^−4^	1.79	2.26 × 10^−6^
*DYRK1B*	7.72	6.62 × 10^−3^	0.84	5.46 × 10^−3^
*EPHB2*	6.86	2.97 × 10^−5^	0.67	1.17 × 10^−6^
*FNIP2*	9.26	1.26 × 10^−3^	1.42	2.30 × 10^−7^
***GOLPH3***	**7.72**	**1.04** × **10^−3^**	**1.13**	**5.49** × **10^−3^**
*KDELC2*	2.54	9.22 × 10^−3^	1.31	1.86 × 10^−4^
*KDM4C*	4.54	2.38 × 10^−3^	0.9	2.01 × 10^−3^
***LAMTOR2***	**15.43**	**4.79** × **10^−4^**	**1.19**	**3.80** × **10^−5^**
*LYSMD2*	12.35	2.18 × 10^−3^	1.29	8.24 × 10^−5^
*MSMO1*	8.42	1.40 × 10^−3^	0.62	3.35 × 10^−3^
***NCKAP1L***	**4.94**	**9.09** × **10^−3^**	**1.79**	**4.35** × **10^−9^**
*NUDT4*	5.15	6.35 × 10^−3^	1.74	1.93 × 10^−6^
*PCDHA8*	4.98	3.35 × 10^−3^	0.78	9.26 × 10^−3^
*PLLP*	12.35	1.99 × 10^−3^	0.74	1.80 × 10^−3^
*RCAN1*	1.79	9.22 × 10^−3^	0.8	2.82 × 10^−6^
*RELL1*	10.29	6.13 × 10^−3^	0.77	1.46 × 10^−4^
***RFFL***	**15.43**	**9.17** × **10^−4^**	**0.76**	**2.74** × **10^−8^**
*TMEM14C*	12.35	3.79 × 10^−4^	1.31	1.17 × 10^−6^
*TOPBP1*	10.29	5.03 × 10^−3^	1.12	3.00 × 10^−3^
*TPK1*	1.21	8.75 × 10^−3^	1.37	1.37 × 10^−6^
*TTLL5*	2.98	3.20 × 10^−3^	0.74	1.29 × 10^−8^

Rare variant analysis was performed in the UK Biobank comparing males who performed more than 6 h of ‘strenuous exercise’ and/or more than 12 h of ‘other exercise’ per week (*n* = 3615) to zero ‘strenuous sport’ or ‘other exercise’ per week (*n* = 107 200). Significance was calculated using Firth logistic regression; OR and FDR corrected *P*-values are provided. For the same genes we provide fold change and FDR corrected *P*-values for differential expression in ALS versus control cervical spinal cord. Genes associated with mTOR signalling are highlighted in bold. ALS = amyotrophic lateral sclerosis; LOF = loss of function; OR = odds ratio; FDR = false discovery rate; FC = fold change; mTOR = mammalian target of rapamycin.

In females (2133 extreme exercisers versus 127 061 sedentary individuals) the same genetic analysis identified *n* = 209 genes associated with performance of extremely strenuous exercise (Firth logistic regression, FDR < 0.01; Supplementary Table 3). Female extreme exercise genes are also significantly enriched with genes that are differentially expressed in ALS compared with control cervical spinal cord (Fisher exact test, *P* = 0.01, overlap of 53 genes), but pathways related to mTOR signalling are not significantly enriched in the overlapping genes (adjusted *P* > 0.05). We did not observe a significant overlap between known ALS risk genes and female extreme exercise genes.

To gain further mechanistic insight we examined the directional change of transcription in ALS spinal cord^26^ of mTOR signalling genes that are genetically inhibited in males who perform extreme exercise. It is important to realize that the LOF mutations within mTOR signalling genes that we observed in extreme exercisers are sufficiently rare to assume that they are absent in the vast majority of the 154 subjects who contributed to the spinal cord transcriptome study.^26^ Three genes implicated in positive regulation of mTOR signalling are upregulated ALS patient spinal cord tissue (Fig. 5B–D), which could be consistent with a compensatory response to disease onset, assuming that the function of these genes is neuroprotective.^13^ In extreme exercisers carrying LOF variants within these genes, this protective response would be impossible. Another mTOR signalling gene, *RFFL* (Fig. 5E), is downregulated in ALS spinal cord.

## Discussion

Understanding the cause of ALS is crucial for the development of strategies for disease prevention. The majority of ALS is thought to result from gene–environment interactions. It follows that identified environmental risk factors are likely to apply only to individuals with a specific genetic background, and therefore any effort to prevent disease by reducing environmental exposure needs to be targeted. For physical exercise this is particularly crucial because we know that exercise in general confers considerable benefits to physical and mental health, and a large proportion of the population fails to meet the recommended thresholds of exercise per week.^31^ For this reason, a generalized recommendation regarding levels of exercise should be avoided.

The measurements of physical exercise we have employed: indirect measurement via genetic liability, a retrospective case-control questionnaire-based measure of lifetime exercise and a prospective questionnaire in a population-scale dataset, all have different advantages and disadvantages, which was the reason we chose to employ all three. We have provided multiple lines of evidence to suggest that the previously reported relationship between leisure-time physical exercise and ALS^16^ may apply specifically to males. Moreover, our results suggest that a significant part of the risk may be contributed by males performing the highest quantities of frequent or strenuous physical activity. To understand this better we performed a genetic study of the top 5% of exercisers by frequency or intensity; henceforth we shall refer to this as ‘extreme exercise’. It is possible that the sex differences we observed are a by-product of the fact that there are fewer females who perform extreme exercise in all of our datasets; this is particularly true of our study of historical leisure-time physical activity where there is a comparable inverse relationship between physical activity and age of ALS symptom onset in females and males, although the relationship in females is not statistically significant (Fig. 3A and B).

Previous studies support the hypothesis that extreme exercise may be a contributor to the risk of ALS. A study of 212 246 individuals who took part in the annual long-distance cross-country skiing competition (Vasaloppet) from 1989 to 2010 in Sweden^32^ found that the fastest skiers had a 4-fold increased risk and those who participated in more than four races had a 3-fold higher risk of ALS compared with 508 176 non-skiers; in contrast slower skiers had less than half the risk of ALS compared with non-skiers. This led the authors to suggest that physical activity in general is protective against ALS but frequent, strenuous physical activity could be a risk factor. Similarly, we previously used MR to causally link leisure-time physical activity to risk of ALS but we did not identify a significant link with other forms of physical activity including accelerometer measured movement in general.^16^ Finally, a study using LD score regression found that light physical activity, including walking for pleasure and light DIY were associated with reduced ALS risk, while moderate intensity physical activity was associated with a higher risk of ALS.^33^

It is important to consider what biological mechanism could link extreme exercise and ALS risk. Indeed, in other contexts exercise has been shown to be neuroprotective.^34^ In animal models of ALS it has been shown that activation of mTOR signalling via metabotropic cholinergic receptor activation at the neuromuscular junction (NMJ) protects MN from the development of ALS via changes in neuronal excitability.^13^ This provides a mechanism whereby exercise—leading to NMJ activity—could be neuroprotective if mTOR signalling is functioning normally (Fig. 5F, top panel). If mTOR signalling is inhibited, then the same NMJ activity could conceivably be harmful via a failure of neuroprotection (Fig. 5F, bottom panel). In this study we present evidence that mTOR signalling is genetically inhibited in a proportion of males who perform extreme exercise. Similarly, even without genetic inhibition, extreme exercise is feasibly associated with hypoxia and persistent energetic stress, which can also inhibit mTOR signalling.^35,36^ Overall, we suggest that individuals performing the most extreme levels of exercise may be selectively vulnerable to its effects.

We have identified a genetic link between reduced mTOR signalling and extreme exercise in males but the mechanism is unknown. Pharmacological inhibition of mTOR signalling is associated with improvements in cardiac function,^37^ which could feasibly facilitate exercise. Indeed inhibition of mTOR signalling with rapamycin is a popular longevity treatment, and rapamycin extends lifespan more in females than males.^38^ This is consistent with our data, and could suggest that any neurotoxicity resulting from pharmacological mTOR inhibition is reduced in females, perhaps via the neuroprotective effect of oestrogen.^39,40^ Indeed, in other tissues oestrogen is an activator of mTOR signalling.^41,42^

The regulation of mTOR signalling is complex and a detailed consideration is beyond the scope of this study. It is noteworthy that mTOR signalling genes, which are genetically inhibited in those who engage in extreme exercise in a way that may predispose to ALS, are generally upregulated in the spinal cord of ALS patients. This could be consistent with a neuroprotective response to symptom onset in the majority of patients who do not carry LOF mutations within mTOR signalling genes. However, one mTOR signalling gene (*RFFL*) was genetically inhibited in those who engage in extreme exercise and downregulated in ALS spinal cord (Fig. 5E). RFFL ubiquitinates p53, thereby targeting it for degradation and thus inhibiting p53 signalling.^43^ p53 signalling has been specifically associated with MN loss in ALS^44^ suggesting that RFFL LOF could be a cause of disease. Rapamycin has also been observed to phenocopy p53 signalling activation.^45^ Interestingly, observed sex-specific differences in p53 signalling suggest that this pathway may be constitutively more active in males.^46^

In conclusion, we provide evidence that ALS is linked to extreme exercise in males, potentially via inhibition of mTOR signalling. The beneficial effects of mTOR inhibition on longevity could therefore be associated with an increase in MN vulnerability in the face of an environmental factor predisposing to ALS such as physical activity. Our observations might also explain why non-extreme exercise, which is not associated with mTOR inhibition, can be neuroprotective via activation of mTOR signalling at the NMJ. mTOR inhibition and exercise are frequently combined in the practice of ‘anti-ageing’ but our work suggests that caution may be necessary to avoid potential harm. We note that rapamycin has actually been proposed as a treatment for ALS due to its immunomodulatory effects, but in a recent negative clinical trial,^47^ serum and CSF neurofilament^48^ was significantly elevated after treatment with rapamycin compared with placebo, and this difference resolved after rapamycin treatment ceased. The authors suggest that this is due to a direct action of rapamycin to increase translation of neurofilament, but an alternate interpretation could be that rapamycin negatively impacts neuronal health leading to increased release of neurofilament from dying neurons. This provides motivation for further *in vivo* modelling of the effect of exercise, with and without rapamycin treatment, combined with a longitudinal study of MN health.

An important caveat to our work is that we have not performed an individual-level prospective analysis, which is the gold standard for the prediction and verification of gene–environment interactions. Our work provides motivation to focus future study on the interaction between physical activity and the risk of ALS on males performing extreme exercise, but should not be used to advise reduced physical activity in the general population.

## Supplementary Material

awaf235_Supplementary_Data

## Data Availability

GWAS summary statistics are available and linked from the original manuscripts ('Materials and methods' section). UKB data are available by application to the UKB: https://www.ukbiobank.ac.uk/enable-your-research/apply-for-access. Transcriptome profiling of ALS patient and control spinal cord is available from the original publication.^26^
